# A cellular platform for the evaluation of immune checkpoint molecules

**DOI:** 10.18632/oncotarget.17615

**Published:** 2017-05-04

**Authors:** Sabrina Jutz, Annika Hennig, Wolfgang Paster, Ömer Asrak, Dejana Dijanovic, Florian Kellner, Winfried F. Pickl, Johannes B. Huppa, Judith Leitner, Peter Steinberger

**Affiliations:** ^1^ Division of Immune Receptors and T cell Activation, Institute of Immunology, Center for Pathophysiology, Infectiology and Immunology, Medical University of Vienna, Vienna, Austria; ^2^ Department of Molecular Immunology, Immune Recognition Unit, Institute for Hygiene and Applied Immunology, Center for Pathophysiology, Infectiology and Immunology, Medical University of Vienna, Vienna, Austria; ^3^ Division of Cellular Immunology and Immunohematology, Institute of Immunology, Center for Pathophysiology, Infectiology and Immunology, Medical University of Vienna, Vienna, Austria

**Keywords:** T cell activation, NF-κB, reporter cell lines, Jurkat, immune checkpoints

## Abstract

Blockade of the T cell coinhibitory molecules CTLA-4 and PD-1 has clinical utility to strengthen T cell responses. In addition to these immune checkpoints an ever-growing number of molecules has been implicated in generating coinhibitory signals in T cells. However, investigating coinhibitory molecules in primary human cells is complicated by the restricted expression and promiscuity of both coinhibitory receptors and their ligands. Here we have evaluated the potential of fluorescence-based transcriptional reporters based on the human Jurkat T cell line in conjunction with engineered T cell stimulator cell lines for investigating coinhibitory pathways. CTLA-4, PD-1, TIGIT, BTLA and 2B4 expressing reporter cells were generated and activated with T cell stimulator cells expressing cognate ligands of these molecules.

All accessory molecules tested were functional in our reporter system. Engagement of CTLA-4, PD-1, BTLA and TIGIT by their ligands significantly inhibited T cell activation, whereas binding of 2B4 by CD48 resulted in enhanced responses. Mutational analysis revealed intracellular motifs that are responsible for BTLA mediated T cell inhibition and demonstrates potent reporter inhibition by CTLA-4 independent of cytoplasmic signaling motifs. Moreover, considerably higher IC50 values were measured for the CTLA-4 blocker Ipilimumab compared to the PD-1 antibody Nivolumab.

Our findings show that coinhibitory pathways can be evaluated in Jurkat-based transcriptional reporters and yield novel insights on their function. Results obtained from this robust reductionist system can complement more time consuming and complex studies of such pathways in primary T cells.

## INTRODUCTION

Accessory signals play a decisive role in the outcome of T cell responses to antigen [1]. Full T cell activation depends on the engagement of costimulatory receptors like CD28 by their cognate ligands expressed on antigen-presenting cells [2]. T cell coinhibitory receptor-ligand interactions on the other hand function as checkpoints, thereby limiting and terminating T cell responses. It is well established that such pathways are essential to prevent aberrant immune responses and maintain tolerance towards self. There is an ever-growing number of surface receptors that have been described to contribute to T cell coinhibitory processes [3].

Immune checkpoints, however also diminish desired T cell responses and blocking coinhibitory receptors like PD-1 or CTLA-4 was shown to be effective in cancer treatments [4-7]. It is conceivable that additional immune checkpoints also control anti-tumor T cell responses. Following this rationale, a LAG-3 fusion protein as well as an antibody targeting the inhibitory LAG-3 receptor are currently being evaluated in clinical trials [8, 9]. The considerable potential of T cell coinhibitory pathways in cancer immunotherapy has fuelled intense efforts to reveal the molecular modus operandi of T cell-expressed receptors like BTLA, TIGIT, CD96, TIM-3, 2B4 and LAG-3 which have all been implicated in negatively regulating T cell responses upon engagement of their cognate ligands. However, studying the function of these molecules in primary human cells face typically considerable obstacles: expression of these receptors is highly variable and depends on the T cell’s activation and differentiation state and its microenvironment [10]. The use of primary APCs, like dendritic cells (DC), for studying T cell inhibitory pathways is often hampered by low ligand expression and consequently insufficient receptor engagement. In addition, APCs harbour a plethora of coinhibitory ligands, which could potentially have redundant functions. Promiscuity among receptors and ligands makes it difficult to ascribe effects observed in primary cells to distinct receptor ligand interactions. Antibodies or immobilized ligands can be used to efficiently and specifically trigger coinhibitory receptors on primary T cells. However, they might generate signals that are not accurately reflecting those transduced upon physiologic engagement by ligands expressed on the cell surface of accessory cells.

In addition coimmobilization of stimulating (CD3) antibodies and inhibitory ligands on beads bear the risk of experimental artefacts due to out-competition of the activatory antibodies by the coimmobilized proteins [11].

Our current understanding of the biology of coinhibitory pathways is largely based on results obtained from animal models. Studies in mice have yielded valuable insights regarding the functional role of coinhibitory receptors in immunity and blocking such molecules in various cancer models is useful to gauge the potential of immune checkpoint inhibitors for cancer therapy. Moreover, the use of animals transgenic for human coinhibitory receptor ligand pairs allows for the preclinical *in vivo* assessment of therapeutics targeting immune checkpoints. However, some of the constraints described for the use of primary human cells also apply to mouse models, and moreover findings in murine model systems might not always accurately reflect the function of these molecules in human cells.

Studies on transformed T cell lines have given valuable insights into signal transduction processes ensuing engagement of the TCR complex and costimulatory receptors [12-18]. The use of such T cell lines for studying coinhibitory pathways has a large potential to overcome impediments associated with primary human T cells. In particular numerous important aspects relating to human coinhibitory pathways become directly accessible to experimentation. Employing a robust T cell system will not only give rise to reproducible data but will also provide molecular and mechanistic insights into immune checkpoints. Results obtained in such a rather reductionist system are bound to complement observations made in primary human cells and pre-clinical animal models. Furthermore and most importantly, they could not only serve as a guiding principle for more intricate and time-consuming studies but may be easily implemented into a high throughput data platform to screen for agonists or antagonists to immune checkpoints.

Here we have engineered fluorescence-based transcriptional reporters based on the human Jurkat T cell line to express CTLA-4, PD-1, BTLA, 2B4 or TIGIT. T cell stimulator cells expressing the respective ligands for these molecules were used to specifically and physiologically trigger these receptors during T cell receptor engagement. The results of this study demonstrate that our cell line-based platform is a powerful and versatile tool to investigate T cell coinhibitory pathways and reveal novel insight into the function of immune checkpoints.

## RESULTS

### Use of a transcriptional reporter T cell line for the assessment of PD-1 mediated coinhibition

**Figure 1:**
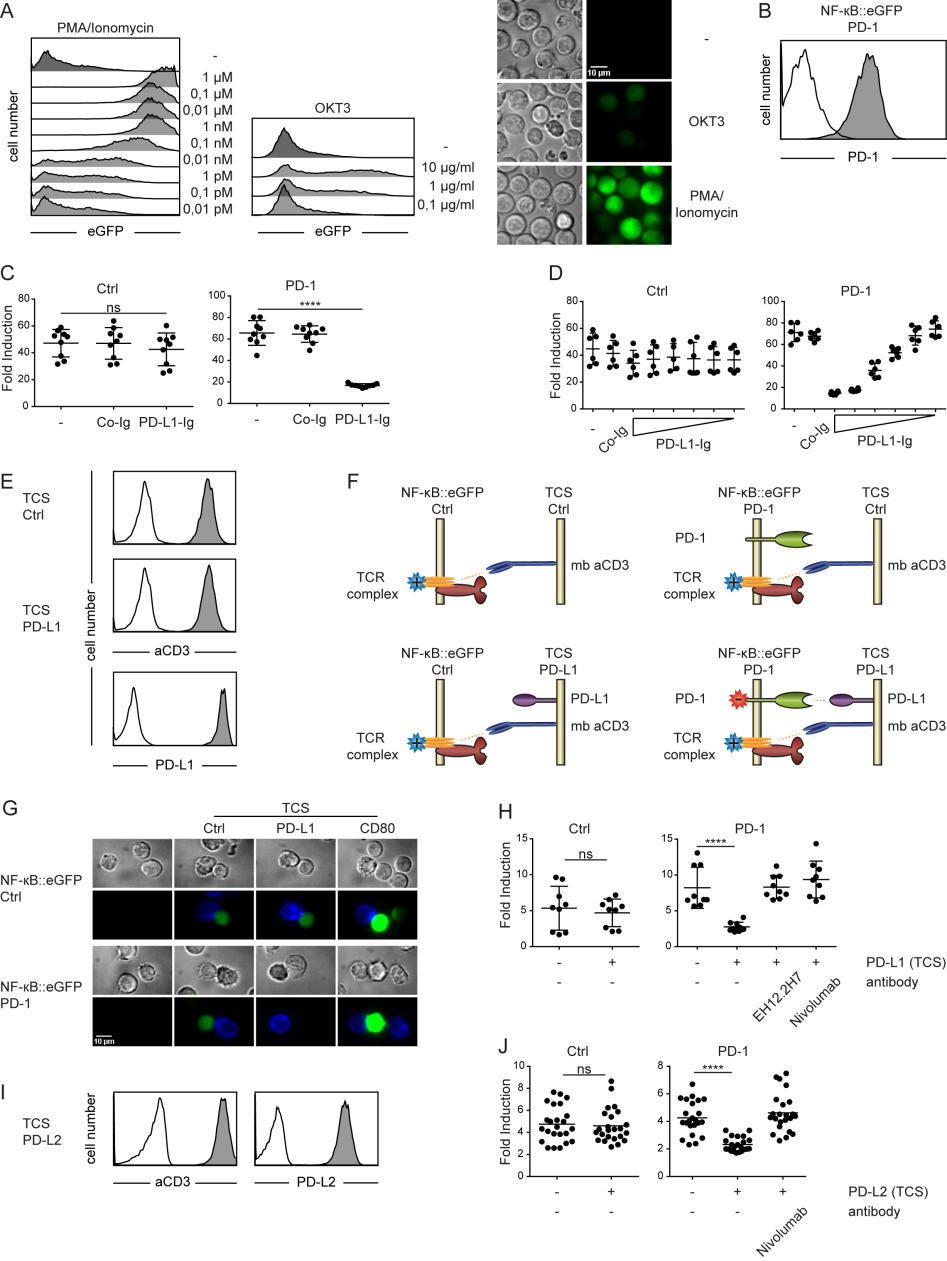
Use of a transcriptional reporter T cell line for the assessment of PD-1 mediated coinhibition **A.** Reporter cells were stimulated with PMA/Ionomycin (left) or cultured on plates where CD3 was immobilized in the indicated concentrations (middle) and eGFP expression was measured *via* flow cytometry. (Right) Shown are microscopy images of reporter cells illustrating their eGFP expression when left untreated (upper panels) stimulated with immobilized CD3 (used at 1 µg/ml, middle panels) or with PMA/Ionomycin (1 µM final concentration; lower panels). **B.** Control reporter cells (open histogram) and PD-1 expressing reporter cells (filled histogram) were stained with a PD-1 antibody and analyzed by flow cytometry. **C.** Control reporters (Ctrl) or reporters expressing PD-1 (PD-1) were stimulated for 24h with immobilized CD3 in presence of co-immobilized control fusion proteins (Co-Ig) or a PD-L1 fusion protein (PD-L1-Ig) as indicated. Results are shown from three independent experiments performed in triplicates. For statistics unpaired *t*-tests were performed (**** *P* ≤ 0.0001; ns *P* > 0.05). **D.** Control reporter (Ctrl) or reporters expressing PD-1 (PD-1) were stimulated for 24h with immobilized CD3 in presence of co-immobilized control fusion proteins (Co-Ig; 10 µg/ml) or a PD-L1 fusion protein (PD-L1-Ig immobilized at concentrations of 20, 10, 5, 2.5, 1.25 and 0.625 µg/ml) as indicated. Results shown are representative for two independent experiments performed in triplicates. **E.** Flow cytometry analysis of TCS. Open histograms: isotype control; filled histograms: reactivity of antibodies to the indicated molecules. **F.** Schematic representation of co-cultures performed in (G and H). **G.** Microscopy images of unstimulated control reporter cells and PD-1 expressing reporter cells and reporters stimulated with TCS-Ctrl, TCS expressing PD-L1 or CD80. TCS are stained with an APC-labelled mCD45 mAb (blue). Reporter gene expression (eGFP) is shown in green. Characterization of TCS-CD80 is shown in Figure 5A. **H.** Control reporter (Ctrl) and PD-1 expressing reporters (PD-1) were stimulated with either TCS or TCS expressing PD-L1, and eGFP expression was measured *via* flow cytometry. PD-1 mAbs (clone EH12.2H7 and Nivolumab, each used at 10 µg/ml) were added as indicated. Results shown are from three independent experiments performed in triplicates. **I.** Flow cytometry analysis of TCS expressing PD-L2. Open histograms: isotype control; filled histograms: reactivity of antibodies to the indicated molecules. **J.** Control reporter (Ctrl) and PD-1 expressing reporters (PD-1) were stimulated with either TCS or TCS expressing PD-L2, and eGFP expression was measured *via* flow cytometry. Nivolumab (10 µg/ml) was added as indicated. Results shown are from eight independent experiments performed in triplicates. **C.**, **D.**, **H.** and **J.** Reporter activation is shown as fold induction (gMFI of TCS stimulated cells/gMFI of unstimulated cells). For statistics unpaired *t*-tests were performed (****P ≤ 0.0001; ns *P* > 0.05).

The human T cell line Jurkat E6.1 was transduced to express a transcriptional NF- κB::eGFP reporter and a clone exerting high sensitivity towards stimulation with PMA/Ionomycin and immobilized anti-CD3 was selected for further use (Figure 1A). PD-1 was expressed in these Jurkat reporter cells and a cell clone that had high and homogenous PD-1 expression was selected for further studies (Figure 1B). PD-1 expressing reporter cells and control reporters were stimulated in the presence of immobilized immunoglobulin fusion proteins representing the extracellular domains of PD-L1 (PDL1-Ig). PDL1-Ig potently inhibited PD-1 reporter activation in a dose-dependent manner (Figure 1C, 1D). In a next set of experiments, T cell stimulator cells (TCS) that coexpress membrane-bound anti-CD3-scFv and high levels of PD-L1 were generated to trigger PD-1 signaling (Figure 1E). Importantly, the availability of reporters lacking PD-1 and TCS expressing membrane-bound anti-CD3 single chain antibody fragment but not PD-L1 allow to assess the effects of PD-1-PD-L1 interaction in a well-controlled system (Figure 1F). Fluorescence microscopy revealed strongly reduced reporter gene expression in PD-1 reporter cells compared to that observed in control reporter cells stimulated in presence of PD-L1. In contrast, stimulation with TCS expressing CD80 greatly enhanced eGFP expression in both reporter cell lines (Figure 1G). Flow cytometric analysis confirmed that PD-1 reporter activation was strongly inhibited by the presence of PD-L1 and furthermore demonstrated that this effect was fully reverted in the presence of blocking PD-1 antibodies (Figure 1H). Stimulation of PD-1 reporter cells with TCS expressing PD-L2 also resulted in a strongly reduced reporter activation (Figure 1I, 1J). These experiments demonstrate that engagement of PD-1 by its cognate ligands results in an efficient and dose-dependent downregulation of NF-κB activity in our human Jurkat reporter T cell line.

### Engagement of TIGIT by CD112, CD113 and CD155 inhibits T cell reporter activation

**Figure 2:**
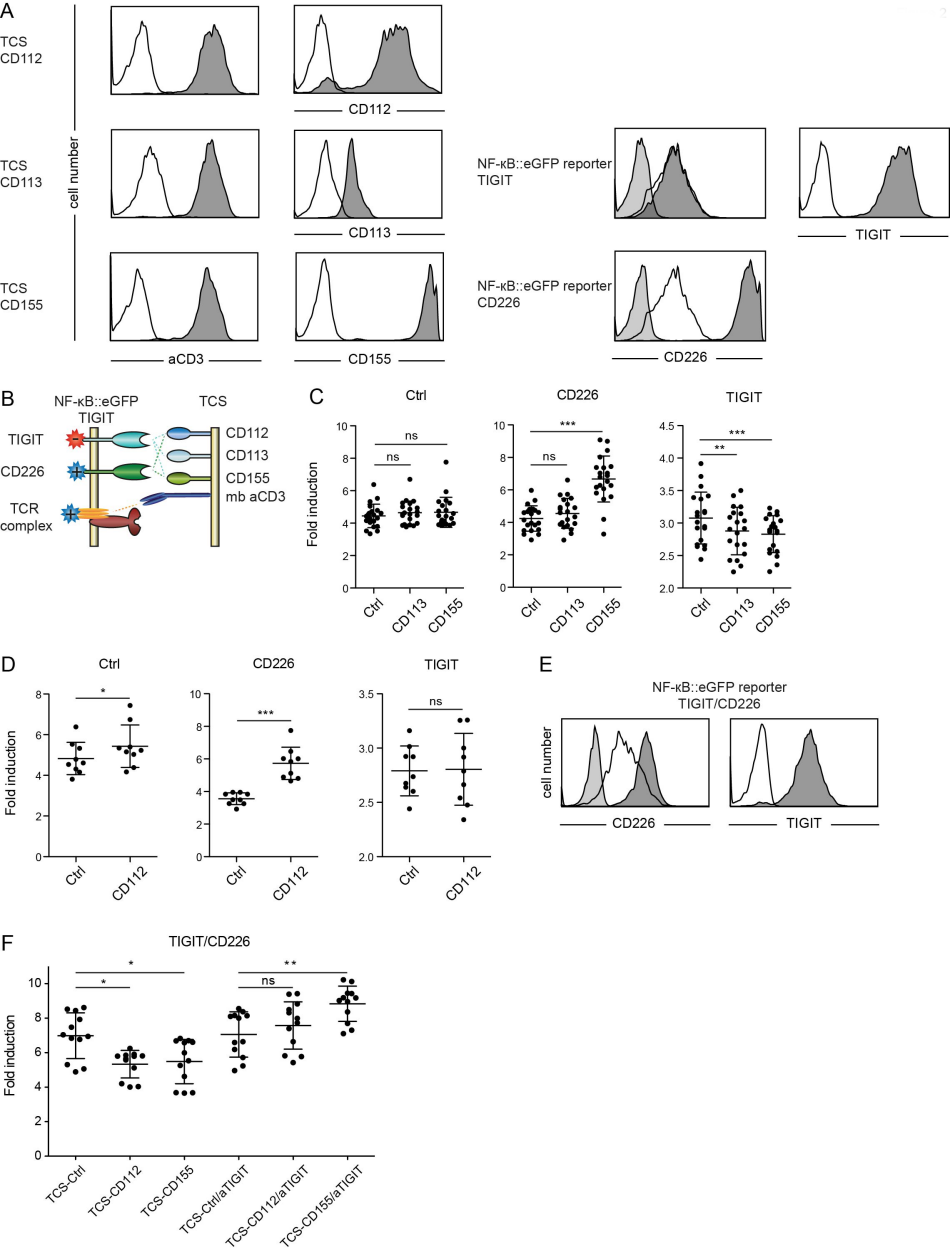
Evaluation of TIGIT and CD226 **A.** Flow cytometry analysis of TCS and NF-κB::eGFP reporter cells. Open histograms: control cells (reporter) or isotype control (TCS); filled histograms (light grey): isotype control; filled histograms (dark grey): expression of indicated molecules on TCS or reporter cells. **B.** Schematic representation of receptors and ligands evaluated. **C.** Control reporters (Ctrl) and reporters overexpressing CD226 or TIGIT were stimulated with TCS as indicated on the x-axis, and eGFP expression was measured *via* flow cytometry. Results from 21 independent experiments are shown. **D.** Control reporters (Ctrl) and reporters overexpressing CD226 or TIGIT were stimulated with control TCS (Ctrl) or TCS expressing CD112, and eGFP expression was measured *via* flow cytometry. Results obtained from nine independent experiments are shown. **E.** Flow cytometry analysis of NF-κB::eGFP reporter cells co-expressing CD226 and TIGIT at comparable levels. Open histograms: expression of indicated molecules on control reporter cells; filled histograms (light grey): isotype control; filled histograms (dark grey): expression of indicated molecules. **F.** Reporter cells shown in (E) were stimulated with control TCS or TCS expressing CD112 and CD155. A blocking TIGIT mAb was used in a final concentration of 8 μg/ml as indicated. Results shown are from four independent experiments performed in triplicates. **C.**, **D.** and **F.** Reporter activation is shown as fold induction (gMFI of TCS stimulated cells/gMFI of unstimulated cells). Each data point represents the reporter activity of an independent experiment and mean and standard deviation are also depicted. Statistics by one-way ANOVA, followed by Tukey’s multiple comparison post test (* *P* ≤ 0.05; ** *P* ≤ 0.01; *** *P* ≤ 0.001; ns *P* > 0.05).

The costimulatory CD226 (DNAM-1) and the coinhibitory receptor TIGIT (WUCAM) bear similarities with CD28 and CTLA-4 and they also share two ligands, CD112 (Poliovirus receptor-related 2; PVRL2 also known as nectin-2) and CD155 (Poliovirus receptor, PVR). Binding studies performed by Yu et al., indicate that in addition to CD112 and CD155 also CD113 (Poliovirus receptor-related 3, PVRL3 also known as nectin-3) ligates TIGIT, whereas no evidence for such an interaction was found in another study [19, 20]. We wanted to investigate these pathways in our system to understand the role of individual ligands in mediating activation or inhibition *via* CD226 and TIGIT, respectively. Reporter cells expressing TIGIT or CD226, and TCS expressing CD112, CD113 or CD155 were generated and expression of the introduced molecules was confirmed by flow cytometry. These experiments revealed that our Jurkat cells endogenously express moderate levels of CD226. The low binding signal obtained upon analysis of CD113 expression is likely due to a weak reactivity of the antibody employed (Figure 2A). In stimulation experiments of reporters with control TCS and TCS expressing CD113 or CD155, a costimulatory effect of CD155 was only observed in reporters overexpressing CD226. Moreover, compared to stimulation with control TCS the response of TIGIT reporters stimulated with TCS expressing CD113 or CD155 was significantly lower (Figure 2B, 2C). This demonstrates that CD113 and CD155 are both functional ligands for the inhibitory TIGIT. Coculture experiments with TCS expressing CD112 indicated that the low endogenous expression of CD226 on our Jurkat reporter cells is already sufficient to mediate CD226-CD112 costimulation (Figure 2D). Overexpression of CD226 resulted in stronger costimulation, whereas the expression of TIGIT abrogated the costimulatory effect of CD112 (Figure 2D). We also analyzed the effect of TIGIT on reporter cells expressing higher levels of CD226 (Figure 2E). Stimulation of these cells in presence of CD155 or CD112 resulted in reduced reporter activation indicating that the inhibitory effects of TIGIT override CD226 costimulation (Figure 2F). The presence of a blocking antibody abrogated inhibition mediated by TIGIT engagement (Figure 2F). Thus our results suggest that in cells co-expressing TIGIT and CD226 the inhibitory effect of TIGIT prevails over CD226 costimulation.

### The CD244-CD48 axis costimulates T cell reporter activation

**Figure 3:**
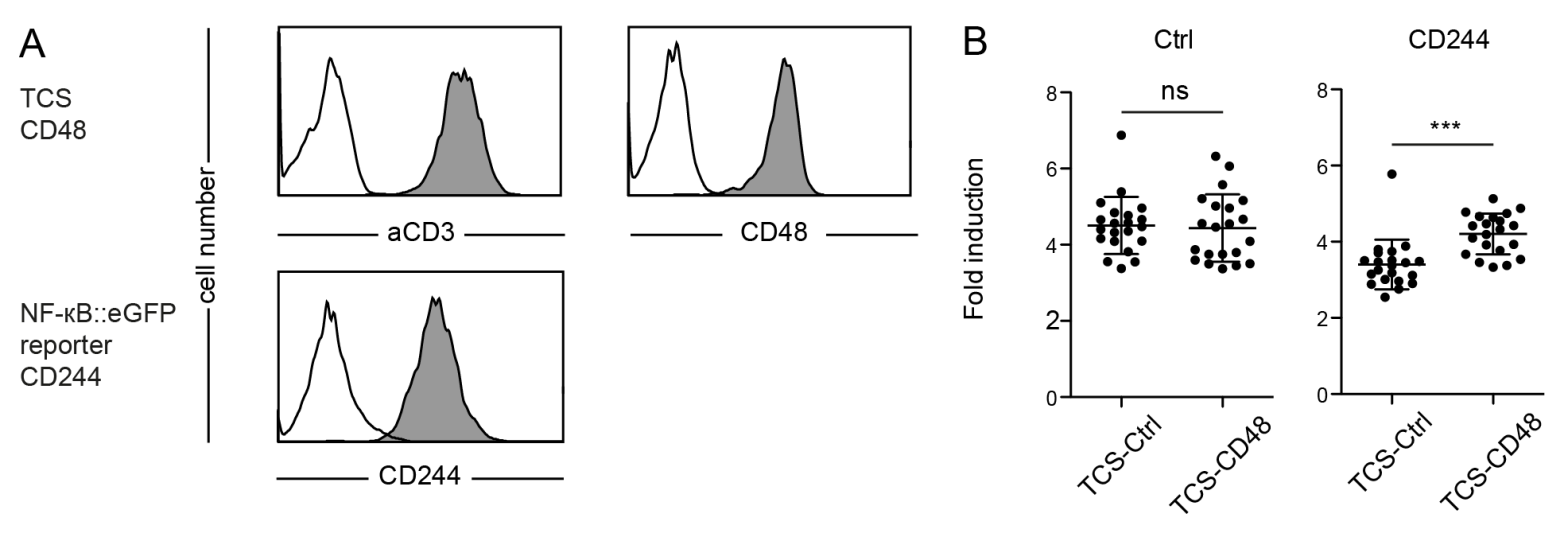
Evaluation of reporter cells expressing CD244 **A.** Flow cytometry analysis of TCS and NF-κB::eGFP reporter cells. Open histograms: control cells (reporter) or isotype control (TCS); filled histograms: Expression of indicated molecules on TCS or reporter cells. **B.** Control reporters (Ctrl) and reporters expressing CD244 (CD244) were stimulated with TCS as indicated on the x-axis and eGFP expression was measured *via* flow cytometry. Reporter activation is shown as fold-induction (gMFI of TCS stimulated cells/gMFI of unstimulated cells). 21 independent experiments were performed and each data point represents the reporter activity of an independent experiment and mean and standard deviation are also shown. Statistics by one-way ANOVA, followed by Tukey’s multiple comparison post test (*** *P* ≤ 0.001; ns *P* > 0.05).

2B4 (CD244) is a member of the CD2 superfamily expressed on NK cells and subsets of T cells. In human T cells activating as well as inhibitory functions have been ascribed to this receptor [21-23]. In order to investigate the effect of CD244 in our system we generated CD244 expressing reporter cells and T cell stimulator cells expressing CD48 (Figure 3A). Whereas the capacity of control TCS and TCS over-expressing CD48 to stimulate control reporter cells did not differ, we observed that the presence of CD48 led to a significantly higher stimulation of CD244 expressing reporter cells (Figure 3B). These results clearly demonstrate a costimulatory effect of the CD244 - CD48 axis in our reporter system.

### Murine coinhibitory receptors are functional and inhibit human T cell reporters

**Figure 4:**
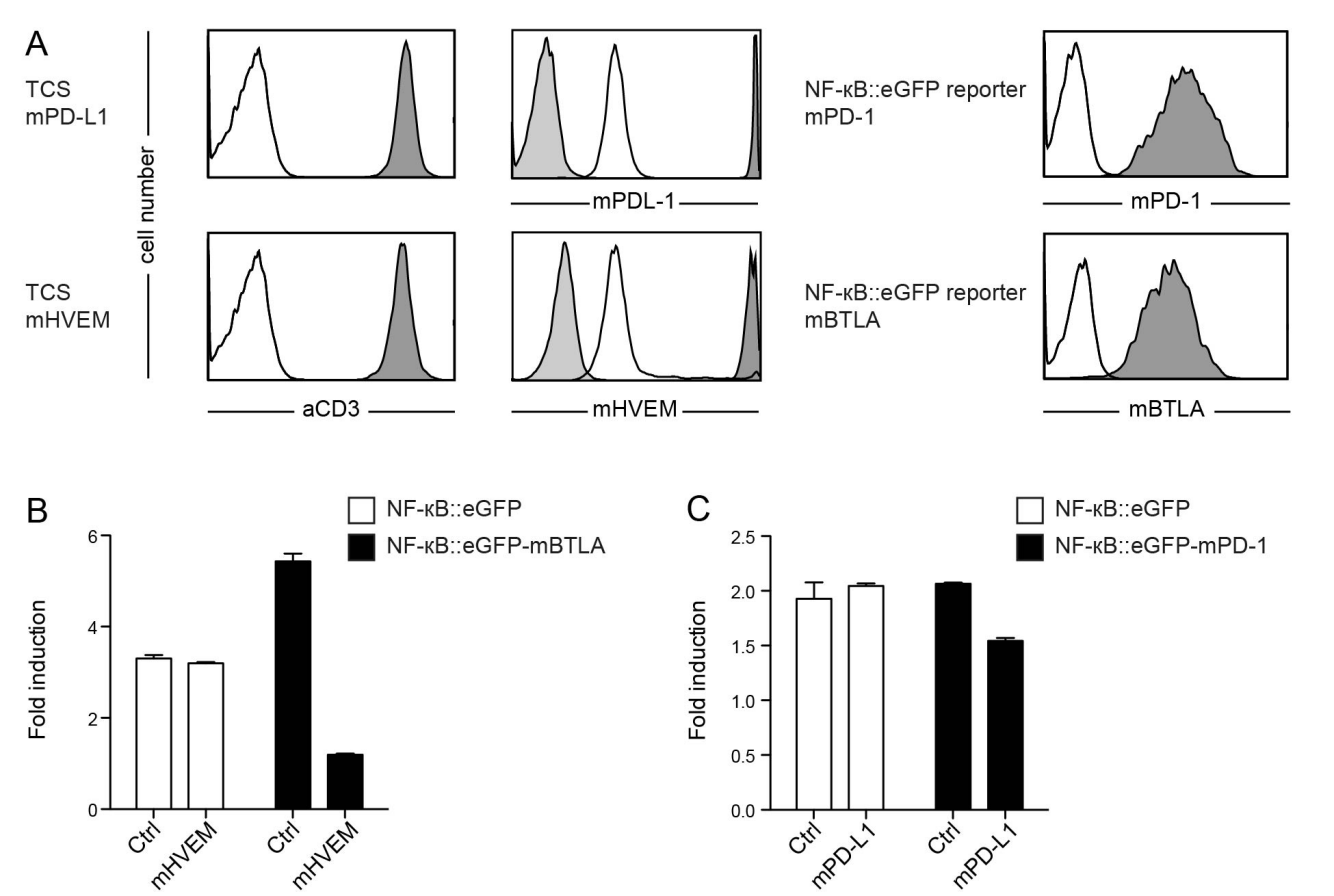
Evaluation of reporter cells expressing murine BTLA or murine PD-1 **A.** Flow cytometry analysis of TCS and NF-κB::eGFP reporter cells. Open histograms: control cells; filled histograms (light grey): isotype control; filled histograms (dark grey): expression of indicated molecules on TCS or reporter cells. **B.** Control reporter cells (Ctrl) and reporter cells expressing mBTLA were stimulated with control TCS or TCS overexpressing murine HVEM as indicated on the x-axis, and eGFP expression was measured *via* flow cytometry. **C.** Control reporter cells (Ctrl) and reporters cells expressing mPD-1 were stimulated with control TCS or TCS overexpressing murine PD-L1 as indicated on the x-axis, and eGFP expression was measured *via* flow cytometry. **B.**+ **C.** Mean and standard deviation from duplicates is shown. Reporter activation is shown as fold-induction (gMFI of TCS stimulated cells/gMFI of unstimulated cells). Experiments are representative for four independently performed experiments.

The effect of immune checkpoint inhibition is widely studied in murine models. Hence, we sought to test whether our platform can also be used for the assessment of murine T cell coinhibitory pathways. To this end we generated a set of TCS expressing either murine PD-L1 or HVEM, as well as NF-κB::eGFP reporter cells expressing murine PD-1 or murine BTLA (Figure 4A). For both pathways, we observed an inhibition of NF-κB activation upon engagement of murine PD-1 or BTLA by their respective ligand (Figure 4B, 4C). This indicates that the human Jurkat T cell line has utility for assessing murine immune checkpoint molecules.

### Extrinsic effects of CTLA-4 potently inhibit T cell reporter activation

**Figure 5:**
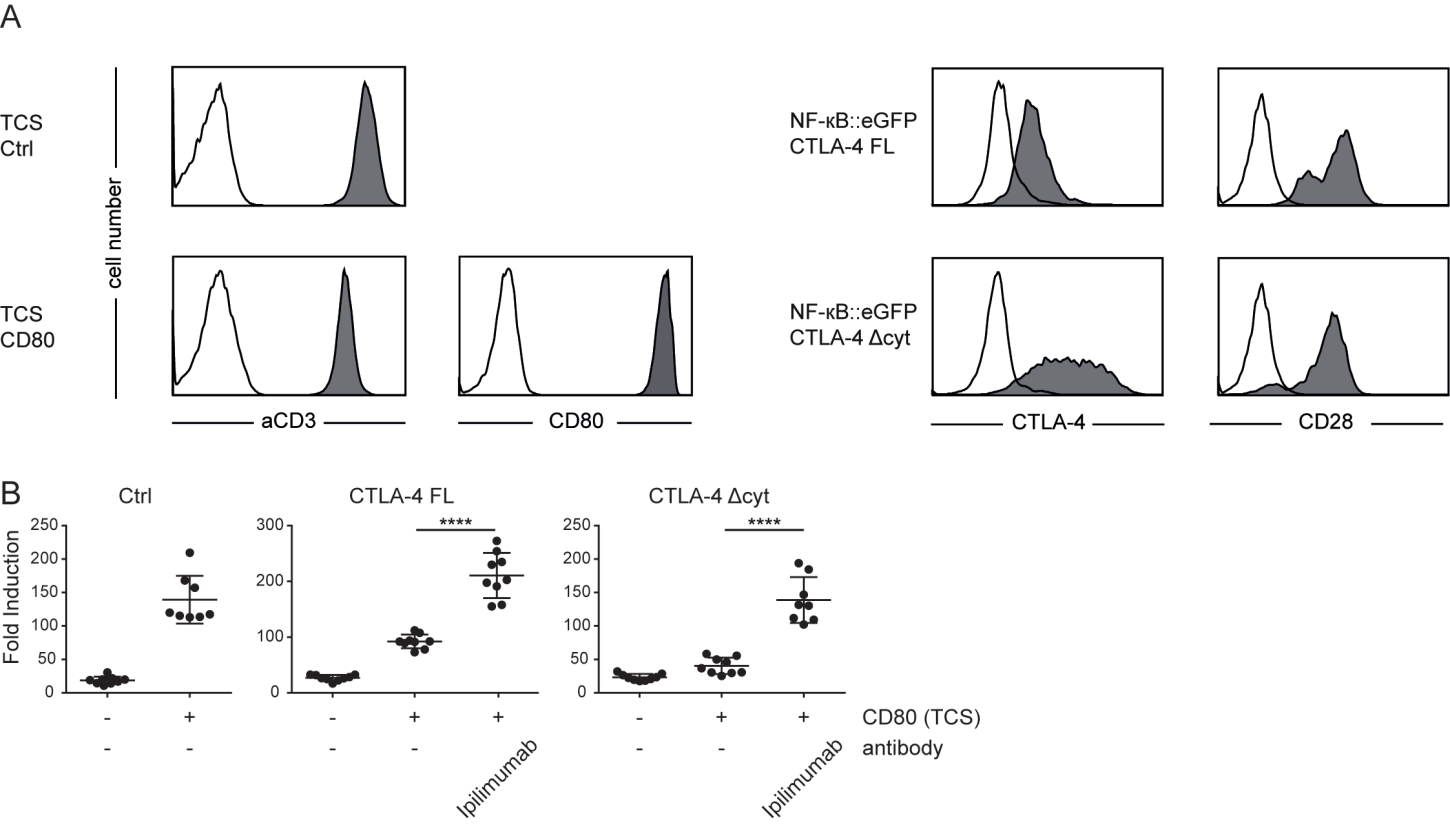
Extrinsic effects of CTLA-4 potently inhibit T cell reporter activation **A.** Flow cytometry analysis of TCS and NF-κB::eGFP reporter cells. Open histograms: control cells (reporter) or isotype control (TCS); filled histograms: Expression of indicated molecules on TCS or reporter cells. **B.** Control reporter (Ctrl) and reporters that express a full-length CTLA-4 (CTLA-4 FL) or a CTLA-4 lacking the cytoplasmic domain (CTLA-4 Δcyt) were stimulated with either TCS or TCS expressing CD80 and eGFP expression in the reporter cells was measured *via* flow cytometry. Ipilimumab (100 µg/ml final concentration) was added as indicated. Results from three independent experiments performed in triplicates are shown. For statistics unpaired *t*-tests were performed (**** *P* ≤ 0.0001; ns *P* > 0.05).

Both extrinsic as well as intrinsic effects have been implicated in contributing to the inhibitory effects of CTLA-4 [24-28]. Our T cell reporters do not express significant amounts of CTLA-4 and can thus be used to study the effects of CTLA-4 and mutants thereof in gain of function experiments. We expressed full length human CTLA-4 as well as a truncated CTLA-4 lacking the entire intracellular domain (CTLA-4 Δcyt) in our reporters and used control TCS and TCS expressing high levels of CD80 for stimulation experiments (Figure 5A). Compared to the control reporter cells, the capacity of CD80 to enhance stimulation of CTLA-4 expressing reporter cells, was strongly reduced (Figure 5B). Moreover, we observed that compared to full length CTLA-4, the variant lacking the cytoplasmic domain had a stronger capacity to inhibit CD80 mediated reporter activation. This is likely due to the higher surface expression of CTLA-4 Δcyt, which will more efficiently prevent the CD80-CD28 interaction. As expected, the presence of the CTLA-4 antibody Ipilimumab completely reverted CTLA-4 effects (Figure 5B). Thus in our reporter T cell system inhibition *via* CTLA-4 can be attributed to extrinsic effects.

### Tyrosine motifs that mediate BTLA-inhibition

**Figure 6:**
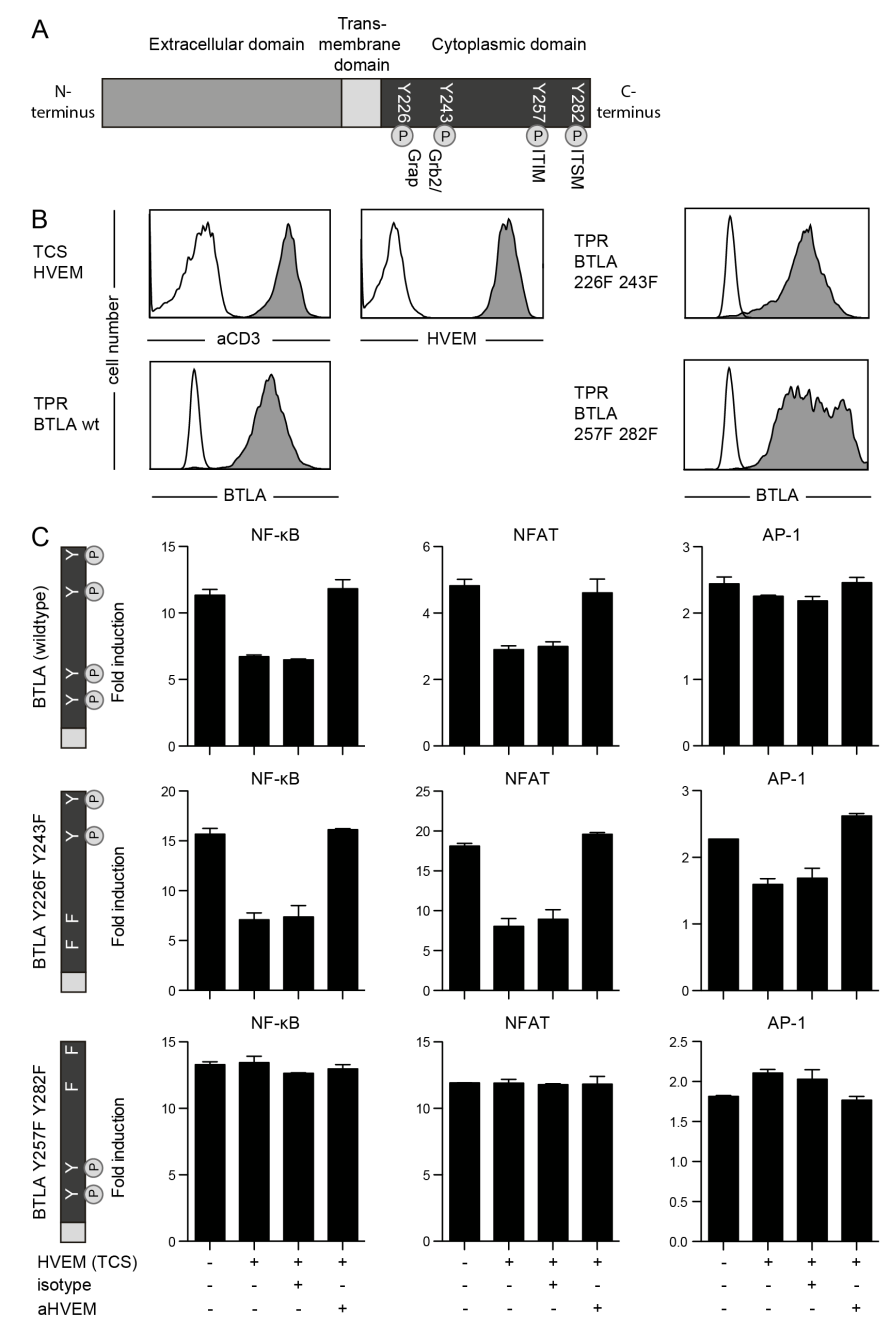
Role of tyrosine motifs in the inhibitory function of BTLA **A.** Schematic illustration of the BTLA molecule showing tyrosine motifs in the cytoplasmic domain. Grap: GRB2-Related Adaptor Protein; ITIM: Immunoreceptor tyrosine-based inhibitory motif; ITSM: Immunoreceptor tyrosine-based switch motif. **B.** TPR reporters expressing wildtype BTLA or BTLA variants harbouring the indicated mutations and TCS were probed with antibodies to the indicated molecules (filled histograms). Open histograms represent reactivity of the respective antibodies with control cells. **C.** TPR reporter cells expressing wildtype BTLA or mutated BTLA were stimulated with control TCS or TCS-HVEM as indicated on the x-axis. Anti-HVEM and isotype control antibody (each used at a final concentration of 5 μg/ml) were added as indicated. Expression of reporter genes for transcriptional activity of NF-κB (CFP), NFAT (eGFP) and AP-1 (mCherry) was measured *via* flow cytometry. Reporter activation is shown as fold-induction (gMFI of TCS stimulated cells/gMFI of unstimulated cells). Mean and standard deviation from duplicates is shown. This experiment is representative for two independently performed experiments.

The role of BTLA as coinhibitory receptor in B and T lymphocytes is well established but it is currently not clear which cytoplasmic motifs mediate these effects. BTLA harbors two Grb2/Grap-sites as well as an ITIM and an ITSM in its cytoplasmic domain (Figure 6A). In order to analyze these tyrosine motifs with respect to their impact on the inhibitory function of BTLA, we generated variants of this molecule in which we mutated tyrosines in the Grb2/Grap-sites or in the ITIM/ITSM motifs to phenylalanine. The wildtype BTLA and the mutated BTLA variants were introduced in recently described triple parameter reporters (TPR), which are also based on the Jurkat T cell line. Flow cytometric analysis revealed comparable expression of BTLA in the three resulting reporter lines (Figure 6B). The TPR allow to simultaneously assess the activity of three transcription factors, since the expression of CFP, eGFP and mCherry is driven by responsive elements for NF-κB, NFAT and AP-1, respectively [29]. We used TCS expressing high levels of HVEM to engage BTLA on the reporter cells (Figure 6B). Signaling *via* wildtype BTLA led to a reduced activation of NF-κB, NFAT and to a lesser extent also of AP-1. This inhibitory effect could be reversed by the addition of a blocking HVEM antibody. TPR expressing BTLA with mutated Grb2/Grap sites also showed strong inhibition of NF-κB, NFAT and AP-1. By contrast, mutation of the tyrosines in the ITIM and ITSM motifs completely abrogated the inhibitory effects of BTLA, indicating that these motifs play an essential role in the inhibitory signal transduction ensuing engagement of BTLA by its ligand HVEM (Figure 6C).

### Evaluation of immune checkpoint inhibitors

**Figure 7:**
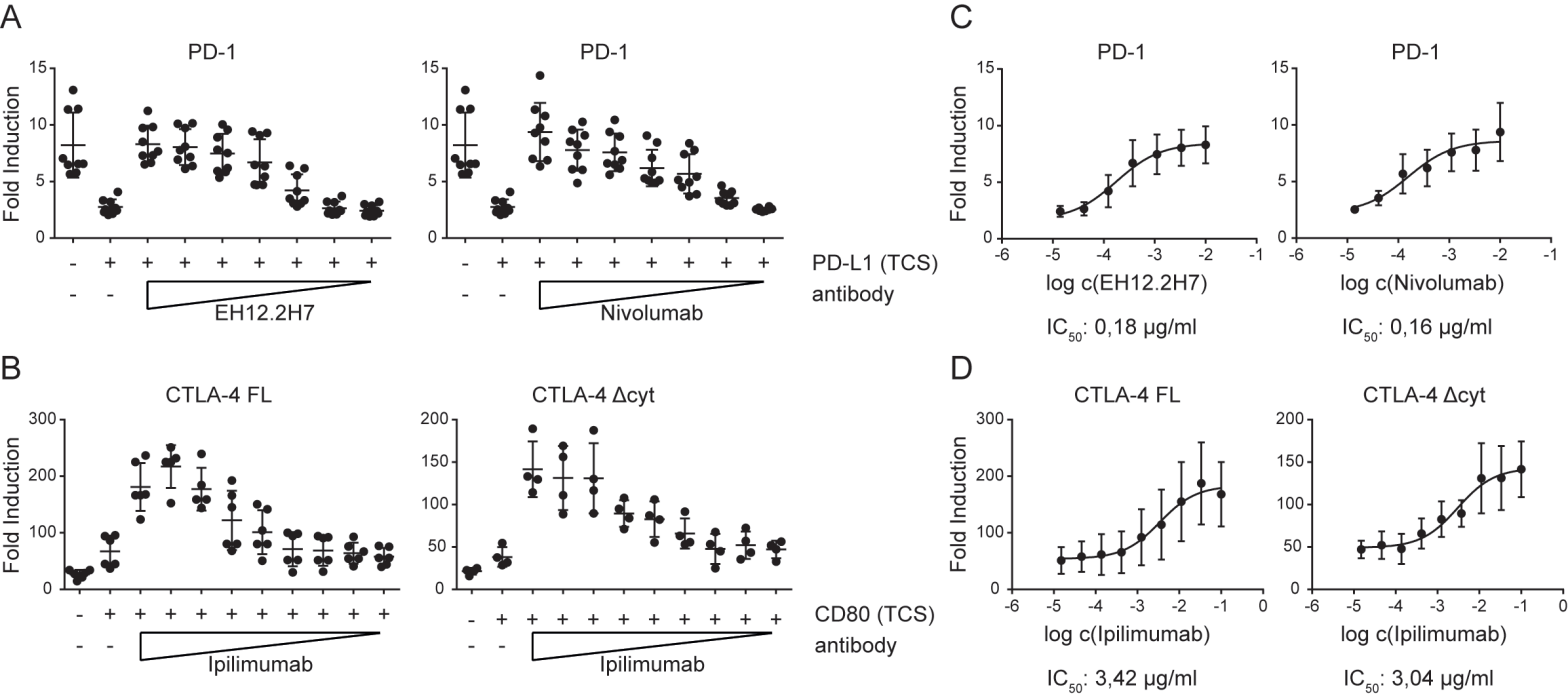
Evaluation of immune checkpoint inhibitors **A.** Control reporters (Ctrl) and PD-1 expressing reporters were stimulated with either TCS or TCS expressing PD-L1, and eGFP expression was measured *via* flow cytometry. PD-1 reporter cells were also stimulated with TCS-PD-L1 in presence of PD-1 antibodies (clone EH12.2H7 and Nivolumab, used at final concentrations of 10, 3.3, 1.1, 0.37, 0.12, 0.04, and 0.014 µg/ml). Results are shown from three independent experiments performed in triplicates. **B.** Control reporter (Ctrl) and reporters that express a full-length CTLA-4 (CTLA-4 FL) or a cytoplasmic deleted CTLA-4 (CTLA-4 Δcyt) were stimulated with either TCS or TCS expressing CD80, and eGFP expression was measured *via* flow cytometry. CTLA-4 FL and CTLA-4 Δcyt were also stimulated with TCS-CD80 in presence of Ipilimumab, used at 100, 33.3, 11.1, 3.7, 1.2, 0.4, 0.14, 0.04 and 0.015 µg/ml. Results are shown from six (FL) or four (Δcyt) independent experiments performed in triplicates. **C.** Half maximal inhibition concentrations (IC_50_) was calculated for the indicated blocking antibodies *via* a nonlinear regression curve followed by a one site competition using GraphPad Prism. **D.** Half maximal inhibition concentration (IC_50_) for Ipilimumab was calculated for CTLA-4 FL and CTLA-4 Δcyt *via* a nonlinear regression curve followed by a one site competition using GraphPad Prism.

Finally we assessed the utility of our reporter system for the evaluation of immune checkpoint inhibitors. To this end we stimulated reporters expressing PD-1 with TCS-PD-L1 in the presence of different concentrations of a commercial PD-1 antibody (EH12.2H7) and the therapeutic Nivolumab also targeting PD-1 (Figure 7A). In additional experiments we used TCS-CD80 to stimulate reporter cells expressing full length CTLA-4 and CTLA-4 Δcyt in presence of the therapeutic anti CTLA-4 antibody Ipilimumab (Figure 7B). IC_50_ calculations indicated a slightly higher efficacy of the therapeutic antibody Nivolumab compared to the commercial anti-PD-1 EH12.2H7 (Figure 7C). Moreover, IC_50_ values calculated for the CTLA-4 antibody Ipilimumab were considerably higher than those calculated for the PD-1 antibody Nivolumab (Figure 7C, 7D). Taken together our data indicate that fluorescence-based T cell reporter systems allow for a cost-effective high-throughput analysis of coinhibitory pathways and the effective evaluation of immune checkpoint inhibitors.

## DISCUSSION

Jurkat T cells are widely used for investigating T cell activation and signaling processes and numerous landmark studies are based on results generated in this cell line as a reductionist model for human T cells [30, 31]. Expression of coinhibitory receptors is low or absent on Jurkat cells even after activation, and to date only a few studies have used Jurkat cells to analyze T cell coinhibitory processes [32-35]. In recent studies we have expressed 4-1BB, PD-1 and BTLA in Jurkat reporter cells and functionally tested these receptors by using T cell stimulator cells which provide the respective ligands to these molecules in the context of TCR-complex driven activation [29, 36]. These experiments indicated that, when ectopically expressed, these T cell costimulatory and coinhibitory receptors are functional in Jurkat T cells. Moreover, we found NF-κB reporter activation potentiated upon 4-1BB engagement and strongly reduced upon BTLA or PD-1 triggering, indicating that the activity of this transcription factor is well suited to gauge both, costimulatory and coinhibitory processes [29]. Hence, we have here analyzed additional coinhibitory pathways in a highly sensitive Jurkat NF-κB-eGFP reporter cell line. Although immobilized recombinant ligands were used to demonstrate a dose-dependent inhibition of reporter cell activation *via* PD-1, we have mainly relied on T cell stimulator cells expressing costimulatory ligands to ensure physiologic triggering of coinhibitory receptors.

In a first set of experiments we have introduced TIGIT in our reporter cells and cocultured the resulting cells as well as control reporter cells with stimulator cells expressing CD112, CD113 or CD155, which have previously been implicated as ligands for this inhibitory receptor. Whereas results by Yu et al. suggested that TIGIT exerts inhibitory effects mainly by inducing tolerogenic DC, other reports indicated that TIGIT inhibits T cells in an intrinsic manner [19, 37]. TIGIT is functional in our reporter cells and our results demonstrate that TIGIT engagement directly inhibits human T cell activation. Moreover our results show that in addition to CD112 and CD155, CD113 can also act as a functional ligand for TIGIT. Importantly, our results indicate that in cells coexpressing TIGIT and CD226 the inhibitory effects of TIGIT overrule CD226 costimulation, suggesting that once expression of TIGIT is induced in T cells they are inhibited in the presence of their common ligands.

When studying 2B4 (CD244) in our reporter system we observed that stimulation of 2B4 expressing reporter cells in the presence of CD48 resulted in enhanced NF-κB reporter activity, indicating that 2B4 might act as a weak costimulatory receptor in human T cells. 2B4 signaling is complex and several pathways are engaged by this receptor which can have both stimulatory and inhibitory activities [38, 39]. Since genes can easily be overexpressed or knocked-down in Jurkat cells these cells might be excellently suited to investigate the compounds that hard-wire human T cells for activating or inhibitory signaling *via* this member of the CD2 superfamily.

T cell coinhibition is widely studied in murine cancer models and humanized mouse models can be used to study immune checkpoint inhibitors *in vivo* [40-42]. We have used murine BTLA and PD-1 to exemplify that murine coinhibitory receptors are functional in our Jurkat system. Thus our system might be useful for the functional analysis of antibodies targeting murine coinhibitory molecules but it might also be used to evaluate the crosstalk between human and murine immune checkpoint pathways, as this could impact on the outcome of studies with “humanized” animals; i.e., mice transgenic for human coinhibitory molecules.

Since Jurkat cells are largely devoid of coinhibitory molecules, mutated or truncated immune checkpoints can be studied in these cells to e.g., analyze the contribution of cytoplasmic motifs to the inhibitory effects of these molecules. Here we have studied modified CTLA-4 and BTLA molecules to shed light on the mode of action of these coinhibitors. CTLA-4 is a well-established coinhibitory receptor but the mechanism of action of this molecule remains surprisingly unclear [43]. A major matter of dispute is the contribution of intracellular signaling *versus* extrinsic effects of CTLA-4 [43]. By comparing a full length CTLA-4 with a variant lacking the entire cytoplasmic domain, we observed that the extracellular domain of CTLA-4 is sufficient to potently suppress T cell activation in a ligand dependent manner. However, these results do not exclude that engagement of CTLA-4 by its natural ligands generates inhibitory signals in T cells. We are currently developing T cell reporter systems based on the CD28 deficient variant of Jurkat line CH7C17 to investigate signal transduction processes induced by CTLA-4 upon engagement by B7 molecules.

Although the role of BTLA as coinhibitory receptor in B and T lymphocytes is well established [44, 45], it is currently not known which signaling motifs transduce inhibitory signals into T cells. Moreover, it was shown that treatment with the phosphatase inhibitor pervanadate but not receptor engagement results in SHP2 recruitment to the cytoplasmic domain of BTLA [46, 47]. Thus it remains unclear whether SHP2, which is well established as mediator of PD-1 inhibition [48, 49], has a physiological role in BTLA function. We found that mutations of tyrosine residues at positions 257 and 282 is sufficient to abrogate inhibitory effects of BTLA. This is in contrast to results obtained in an earlier study by Chemnitz et al., which suggested that the inhibitory effect of BTLA was only disrupted upon mutation of all four tyrosine-based motifs located in the intracellular BTLA-sequence [47]. Our use of the cell-expressed natural ligand HVEM in contrast to engagement of a chimeric BTLA molecule with immobilized antibodies, as done in the study by Chemnitz et al. may explain the observed difference.

In addition to a favorable pharmacokinetic profile and a low immunogenicity, immune checkpoint inhibitors should typically be endowed with a high capability to block the coinhibitory pathway of interest. We have used our Jurkat-TCS-system as a functional readout to determine IC_50_ values for the PD-1 and CTLA-4 antibodies. These experiments demonstrate the utility of this system to assess the potency of immune checkpoint inhibitors. To the best of our knowledge this study is the first to determine the IC_50_ values for Ipilimumab and Nivolumab, which are clinically used to block CTLA-4 and PD-1, respectively, in a cellular system on a functional level. Although similar doses of these drugs are administered in the clinic, our results indicate that these drugs differ considerably in their capacity to block their respective target molecules.

Clinical success of antibodies to CTLA-4 and PD-1 has rekindled the hope for cancer immunotherapy [50]. Several negative checkpoint regulators might restrict the ability of T cells to effectively attack malignant cells. The investigation of each immune checkpoint molecule harbors unique challenges such as multiple ligands in some cases shared with an activating receptor or bidirectional signaling capacities of binding partners that can function both as a receptor and as a ligand. Such high levels of complexity require the availability of reductionist model systems for mechanistic studies. Based on the results presented in this study we propose that transcriptional reporters based on the human T cell line Jurkat are a powerful tool to explore T cell coinhibitory pathways on a functional level.

## MATERIALS AND METHODS

### Antibodies, cell culture and flow cytometry

Bw5147 (short designation: Bw) and Jurkat E6.1 cells were cultured as described previously [51, 52]. The Jurkat E6.1 NF-κB::eGFP reporter T cell line was described previously [53]. Cell lines were tested for absence of mycoplasma (MycoAlert Mycoplasma Detection Kit, Lonza Group AG, Basel Switzerland) and the identity of the Jurkat cells was confirmed by flow cytometric analysis employing a large panel of antibodies to human T cell markers. To confirm expression of ectopically expressed surface molecules, the following PE-conjugated antibodies and appropriate isotype control from Biolegend (San Diego, CA) were used: BTLA (MIH26), HVEM (TR2), mBTLA (8F4), mHVEM (HMHV-1B18), mPD-1 (29F.1A12), mPD-L1 (TY25), PD-L1 (29E.2A3), PD-L2 (24F.10C12), CD155 (SKII.4), CD48 (BJ40), CD80 (2D10). Additionally, a PE-conjugated TIGIT mAb (MBSA43), an APC-conjugated CTLA-4 mAb (14D3) from eBioScience (San Diego, CA), a CD113 mAb (N3.81.6) from Santa Cruz Biotechnology (Santa Cruz, CA) and a PE-conjugated PD-1 mAb (EH12.1) from Becton Dickinson (Franklin Lakes, NJ) were used. For detection of membrane-bound anti-CD3 fragment, a DyLight-649-conjugated goat-anti-mouse IgG (H+L) antibody (Jackson ImmunoResearch, West Grove, PA) or an APC-conjugated CD14 antibody (HCD14, Biolegend) was used. CD112 antibody (5-193) was produced in the laboratory of Otto Majdic, and detected by a PE-conjugated goat-anti-human IgG (Fc-specific) antibody (Jackson ImmunoResearch). Flow cytometry was performed on an LSRFortessa or on a FACSCalibur flow cytometer (Becton Dickinson Immunocytometry System, San Jose, CA), using FACSDiva and CellQuest software, respectively. Data was analyzed with FlowJo (version 10.0.6, Tree Star, Ashland, OR) and Graphpad Prism (version 5, GraphPad Software, Inc., La Jolla, CA).

### Generation of cell lines

T cell stimulator cells (short designation: TCS) were generated as described previously [54]. Briefly, Bw cells were transduced to stably express an anti-human CD3 single chain fragment anchored to the cell membrane *via* a human CD14 stem (CD5L-OKT3scFv-CD14) and thereby providing signal 1. TCS expressing high levels of HVEM, murine HVEM (mHVEM), PD-L1, murine PD-L1 (mPD-L1), CD155, CD48, CD80, CD112 and CD113 were generated *via* retroviral transduction. Flow cytometry analysis was performed to confirm the expression of these molecules.

JE6.1 cells were retrovirally transduced with a NF-κB::eGFP reporter construct and a clone with low background and expression of high levels of eGFP upon stimulation with PMA and Ionomycin (each used at a final concentration of 100 nM) was selected. The generation of triple parameter reporter cells (short designation: TPR) allowing the simultaneous measurement of NF-κB, NFAT and AP-1, was described previously [29]. Tyrosines comprised in phosphotyrosine motifs of BTLAs cytoplasmic domain were replaced with phenylalanines in order to obtain BTLA-Y226F-Y243F and BTLA-Y257F-Y282F. Reporter cells were retrovirally transduced to express CTLA-4 FL (full length), CTLA-4-Δcyt (lacking the cytoplasmic domain), 2B4, TIGIT, CD226, mPD-1, PD-1 or BTLA (wildtype, Y226F-Y243F, Y257F-Y282F or murine BTLA). Expression of these receptors was confirmed *via* flow cytometry analysis.

### Reporter assays

Reporter cells (5x10^4^ cells/well) were cocultivated with TCS (1-1.5x10^4^ cells/well) for 24 hours. In some experiments, polyclonal aHVEM Ab from R&D Systems (Minneapolis, MN) or TIGIT mAb (clone MBSA43) from eBioScience were added at a final concentration of 8 µg/ml. PD-1 mAb (EH12.2H7) from Biolegend (San Diego, CA), Ipilimumab and Nivolumab were used as indicated.

After 24 hours, cells were harvested and the TCS were stained with an APC-conjugated mCD45.2 antibody. Subsequently, expression of reporter genes (eGFP, CFP, mCherry) were measured *via* flow cytometry. Geometric mean of fluorescence intensity of viable reporter cells (viability was determined according to their FSC/SCC profile and APC^+^TCS were excluded) was used for further analysis. For most experiments, reporter gene induction in response to stimulation was normalized to reporter gene expression in the respective non-stimulated reporters and expressed as fold induction of the geometric mean fluorescence intensity (gMFI).

In experiments with plate-bound CD3 antibodies and immobilized immunoglobulin fusion protein ninety-six-well ELISA plates (Nunc MaxiSorp, eBioScience) were used. Plates were coated directly with a CD3 mAb (OKT3, Ortho Pharmaceutical Corp., Raritan, NJ; Figure 1A) or coated overnight at 4°C with a goat-anti-mouse IgG subclass 2a antibody and a goat-anti-human IgG antibody (Jackson ImmunoResearch) in 3 µg/ml and 10 µg/ml final concentrations respectively in 1xPBS. The following day plates were washed and blocked with cell culture medium for 10 min at room temperature. Plates then were incubated with a mixture of CD3 mAb (OKT3) in a final concentration of 1 µg/ml and an immunoglobulin fusion protein (Co-Ig or PD-L1-Ig) in a final concentration as indicated in the different experiments. These immunoglobulin fusion proteins were generated and expressed as previously described [55]. After 60 min of incubation at room temperature reporter cells (5x10^4^ cells/well) were added and cultured at 37°C, 5% CO_2_. After 24 hours cells were harvested and eGFP expression was measured *via* flow cytometry.

### Microscopy

For image acquisition, reporter cells were either stimulated with PMA/Ionomycin (1 µM final concentration for both, PMA and Ionomycin) or plate bound anti-CD3 (1 µg/ml final concentration) on the one hand, and on the other hand cocultured with TCS as indicated. Unstimulated reporter cells were used as a negative control.

Images were acquired on a Leica DMI4000B microscope (Leica Microsystems; Wetzlar, Germany) using a 63x objective (Leica PLAN 506184) and an Andor iXon Ultra-897 EM-CCD camera (Andor Technologies; Belfast, UK). The system was controlled by MetaMorph software (Molceular Devices; Downingtown, PA). Fluorophores were excited with 488 nm (GFP) or 640 nm laser light (APC). The emission was filtered for GFP (FITC filter) and APC (Cy5 filter) using the Leica Quad-Sedat filter system (set 11532564). Images were processed with the software ImageJ (Version 1.49, National Institute of Health, Washington, DC).

### Statistics

For TIGIT, CD226 and CD244 experiments, median of triplicates +/- standard deviation is shown, whereas mean of duplicates +/- standard deviation was used in experiments with murine molecules and BTLA. Data was normalized to unstimulated cells. For statistical analysis, one-tailed paired *t*-test or one-way ANOVA with Tukey post test were performed (ns not significant, *P ≤ 0.05; **P ≤ 0.01; ***P ≤ 0.001).

For PD-1 and CTLA-4 experiments triplicates +/- standard deviation are shown. Data was normalized to unstimulated cells. For statistical analysis one-tailed paired *t*-tests were performed (ns not significant, ****P ≤ 0.0001).

The half maximal inhibitory concentrations (IC_50_) for Ipilimumab, Nivolumab and PD-1 mAb (EH12.2H7) were calculated by plotting the fold induction of NF-κB activation (eGFP expression) against the log concentration of the indicated antibody (mg/ml) using Graphpad Prism.

## Abbreviations

Bw: Bw5147
Ctrl: control
DC: dendritic cells
Δcyt: lacking cytoplasmic domain
gMFI: geometric mean fluorescence intensity
Grap: Grb2-related adaptor protein
IC_50_: half maximal inhibitory concentration
Ig: immunoglobulin
ITIM: Immunoreceptor tyrosine-based inhibitory motif
ITSM: Immunoreceptor tyrosine-based switch motif
FL: full length
TCS: T cell stimulator cells
TPR: Triple parameter reporter

## References

[1] Leitner J, Grabmeier-Pfistershammer K, Steinberger P (2010). Receptors and ligands implicated in human T cell costimulatory processes. Immunol Lett.

[2] Sharpe AH (2009). Mechanisms of costimulation. Immunol Rev.

[3] Chen L, Flies DB (2013). Molecular mechanisms of T cell co-stimulation and co-inhibition. Nat Rev Immunol.

[4] Hodi FS, O’Day SJ, McDermott DF, Weber RW, Sosman JA, Haanen JB, Gonzalez R, Robert C, Schadendorf D, Hassel JC, Akerley W, van den Eertwegh AJ, Lutzky J (2010). Improved survival with ipilimumab in patients with metastatic melanoma. N Engl J Med.

[5] Postow MA, Callahan MK, Wolchok JD (2015). Immune Checkpoint Blockade in Cancer Therapy. Journal of clinical oncology.

[6] Marquez-Rodas I, Cerezuela P, Soria A, Berrocal A, Riso A, Gonzalez-Cao M, Martin-Algarra S (2015). Immune checkpoint inhibitors: therapeutic advances in melanoma. Annals of translational medicine.

[7] Callahan MK, Postow MA, Wolchok JD (2014). CTLA-4 and PD-1 Pathway Blockade: Com-binations in the Clinic. Frontiers in oncology.

[8] Shin DS, Ribas A (2015). The evolution of checkpoint blockade as a cancer therapy: what’s here, what’s next?. Curr Opin Immunol.

[9] Legat A, Maby-El Hajjami H, Baumgaertner P, Cagnon L, Abed Maillard S, Geldhof C, Iancu EM, Lebon L, Guillaume P, Dojcinovic D, Michielin O, Romano E, Berthod G (2016). Vaccination with LAG-3Ig (IMP321) and Peptides Induces Specific CD4 and CD8 T-Cell Responses in Metastatic Melanoma Patients–Report of a Phase I/IIa Clinical Trial. Clin Cancer Res.

[10] Baitsch L, Legat A, Barba L, Fuertes Marraco SA, Rivals JP, Baumgaertner P, Christiansen-Jucht C, Bouzourene H, Rimoldi D, Pircher H, Rufer N, Matter M, Michielin O (2012). Extended co-expression of inhibitory receptors by human CD8 T-cells depending on differentiation, antigen-specificity and anatomical localization. PLoS One.

[11] Fuertes Marraco SA, Baumgaertner P, Legat A, Rufer N, Speiser DE (2012). A stepwise protocol to coat aAPC beads prevents out-competition of anti-CD3 mAb and consequent experimental artefacts. J Immunol Methods.

[12] Northrop JP, Ullman KS, Crabtree GR (1993). Characterization of the nuclear and cytoplasmic components of the lymphoid-specific nuclear factor of activated T cells (NF-AT) complex. J Biol Chem.

[13] Wu J, Katzav S, Weiss A (1995). A functional T-cell receptor signaling pathway is required for p95vav activity. Mol Cell Biol.

[14] Kaizuka Y, Douglass AD, Vardhana S, Dustin ML, Vale RD (2009). The coreceptor CD2 uses plasma membrane microdomains to transduce signals in T cells. The Journal of cell biology.

[15] Vandenberghe P, Freeman GJ, Nadler LM, Fletcher MC, Kamoun M, Turka LA, Ledbetter JA, Thompson CB, June CH (1992). Antibody and B7/BB1-mediated ligation of the CD28 receptor induces tyrosine phosphorylation in human T cells. J Exp Med.

[16] Parry RV, Reif K, Smith G, Sansom DM, Hemmings BA, Ward SG (1997). Ligation of the T cell co-stimulatory receptor CD28 activates the serine-threonine protein kinase protein kinase B. Eur J Immunol.

[17] Swamy M, Beck-Garcia K, Hartl FA, Morath A, Yousefi OS, Dopfer EP, Molnar E, Schulze AK, Blanco R, Borroto A, Martin-Blanco N, Alarcon B, Höfer T (2016). A Cholesterol-Based Allostery Model of T Cell Receptor Phosphorylation. Immunity.

[18] Bruyns E, Marie-Cardine A, Kirchgessner H, Sagolla K, Shevchenko A, Mann M, Autschbach F, Bensussan A, Meuer S, Schraven B (1998). T cell receptor (TCR) interacting molecule (TRIM), a novel disulfide-linked dimer associated with the TCR-CD3-zeta complex, recruits intracellular signaling proteins to the plasma membrane. J Exp Med.

[19] Yu X, Harden K, Gonzalez LC, Francesco M, Chiang E, Irving B, Tom I, Ivelja S, Refino CJ, Clark H, Eaton D, Grogan JL (2009). The surface protein TIGIT suppresses T cell activation by promoting the generation of mature immunoregulatory dendritic cells. Nat Immunol.

[20] Stanietsky N, Simic H, Arapovic J, Toporik A, Levy O, Novik A, Levine Z, Beiman M, Dassa L, Achdout H, Stern-Ginossar N, Tsukerman P, Jonjic S (2009). The interaction of TIGIT with PVR and PVRL2 inhibits human NK cell cytotoxicity. Proc Natl Acad Sci U S A.

[21] Yang B, Wang X, Jiang J, Cheng X (2013). Involvement of CD244 in regulating CD4+ T cell immunity in patients with active tuberculosis. PLoS One.

[22] Schlaphoff V, Lunemann S, Suneetha PV, Jaroszewicz J, Grabowski J, Dietz J, Helfritz F, Bektas H, Sarrazin C, Manns MP, Cornberg M, Wedemeyer H (2011). Dual function of the NK cell receptor 2B4 (CD244) in the regulation of HCV-specific CD8+ T cells. PLoS Pathog.

[23] Ezinne CC, Yoshimitsu M, White Y, Arima N (2014). HTLV-1 specific CD8+ T cell function augmented by blockade of 2B4/CD48 interaction in HTLV-1 infection. PLoS One.

[24] Kong KF, Fu G, Zhang Y, Yokosuka T, Casas J, Canonigo-Balancio AJ, Becart S, Kim G, Yates JR, Kronenberg M, Saito T, Gascoigne NR, Altman A (2014). Protein kinase C-eta controls CTLA-4-mediated regulatory T cell function. Nat Immunol.

[25] Schneider H, Downey J, Smith A, Zinselmeyer BH, Rush C, Brewer JM, Wei B, Hogg N, Garside P, Rudd CE (2006). Reversal of the TCR stop signal by CTLA-4. Science.

[26] Wulfing C, Tunbridge HM, Wraith DC (2014). New inhibitory signaling by CTLA-4. Nat Immunol.

[27] Wang CJ, Kenefeck R, Wardzinski L, Attridge K, Manzotti C, Schmidt EM, Qureshi OS, Sansom DM, Walker LS (2012). Cutting edge: cell-extrinsic immune regulation by CTLA-4 expressed on conventional T cells. J Immunol.

[28] Walker LS, Sansom DM (2011). The emerging role of CTLA4 as a cell-extrinsic regulator of T cell responses. Nat Rev Immunol.

[29] Jutz S, Leitner J, Schmetterer K, Doel-Perez I, Majdic O, Grabmeier-Pfistershammer K, Paster W, Huppa JB, Steinberger P (2016). Assessment of costimulation and coinhibition in a triple parameter T cell reporter line: Simultaneous measurement of NF-kappaB, NFAT and AP-1. J Immunol Methods.

[30] Abraham RT, Weiss A (2004). Jurkat T cells and development of the T-cell receptor signalling paradigm. Nat Rev Immunol.

[31] Irles C, Symons A, Michel F, Bakker TR, van der Merwe PA, Acuto O (2003). CD45 ectodomain controls interaction with GEMs and Lck activity for optimal TCR signaling. Nat Immunol.

[32] Tomkowicz B, Walsh E, Cotty A, Verona R, Sabins N, Kaplan F, Santulli-Marotto S, Chin CN, Mooney J, Lingham RB, Naso M, McCabe T (2015). TIM-3 Suppresses Anti-CD3/CD28-Induced TCR Activation and IL-2 Expression through the NFAT Signaling Pathway. PLoS One.

[33] Li Q, Quan L, Lyu J, He Z, Wang X, Meng J, Zhao Z, Zhu L, Liu X, Li H (2016). Discovery of peptide inhibitors targeting human programmed death 1 (PD-1) receptor. Oncotarget.

[34] Black M, Barsoum IB, Truesdell P, Cotechini T, Macdonald-Goodfellow SK, Petroff M, Siemens DR, Koti M, Craig AW, Graham CH (2016). Activation of the PD-1/PD-L1 immune checkpoint confers tumor cell chemoresistance associated with increased metastasis. Oncotarget.

[35] Lee J, Su EW, Zhu C, Hainline S, Phuah J, Moroco JA, Smithgall TE, Kuchroo VK, Kane LP (2011). Phosphotyrosine-dependent coupling of Tim-3 to T-cell receptor signaling pathways. Mol Cell Biol.

[36] Rosskopf S, Jutz S, Neunkirchner A, Candia MR, Jahn-Schmid B, Bohle B, Pickl WF, Steinberger P (2016). Creation of an engineered APC system to explore and optimize the presentation of immunodominant peptides of major allergens. Scientific reports.

[37] Joller N, Hafler JP, Brynedal B, Kassam N, Spoerl S, Levin SD, Sharpe AH, Kuchroo VK (2011). Cutting edge: TIGIT has T cell-intrinsic inhibitory functions. J Immunol.

[38] Vaidya SV, Mathew PA (2006). Of mice and men: different functions of the murine and human 2B4 (CD244) receptor on NK cells. Immunol Lett.

[39] McArdel SL, Terhorst C, Sharpe AH (2016). Roles of CD48 in regulating immunity and tolerance. Clin Immunol.

[40] Ma SD, Xu X, Jones R, Delecluse HJ, Zumwalde NA, Sharma A, Gumperz JE, Kenney SC (2016). PD-1/CTLA-4 Blockade Inhibits Epstein-Barr Virus-Induced Lymphoma Growth in a Cord Blood Humanized-Mouse Model. PLoS Pathog.

[41] Ai M, Curran MA (2015). Immune checkpoint combinations from mouse to man. Cancer Immunol Immunother.

[42] Morton JJ, Bird G, Refaeli Y, Jimeno A (2016). Humanized mouse xenograft models: narrowing the tumor-microenvironment gap. Cancer Res.

[43] Walker LS, Sansom DM (2015). Confusing signals: recent progress in CTLA-4 biology. Trends Immunol.

[44] Sedy JR, Gavrieli M, Potter KG, Hurchla MA, Lindsley RC, Hildner K, Scheu S, Pfeffer K, Ware CF, Murphy TL, Murphy KM (2005). B and T lymphocyte attenuator regulates T cell activation through interaction with herpesvirus entry mediator. Nat Immunol.

[45] Watanabe N, Gavrieli M, Sedy JR, Yang J, Fallarino F, Loftin SK, Hurchla MA, Zimmerman N, Sim J, Zang X, Murphy TL, Russell JH, Allison JP (2003). BTLA is a lymphocyte inhibitory receptor with similarities to CTLA-4 and PD-1. Nat Immunol.

[46] Gavrieli M, Watanabe N, Loftin SK, Murphy TL, Murphy KM (2003). Characterization of phosphotyrosine binding motifs in the cytoplasmic domain of B and T lymphocyte attenuator required for association with protein tyrosine phosphatases SHP-1 and SHP-2. Biochem Biophys Res Commun.

[47] Chemnitz JM, Lanfranco AR, Braunstein I, Riley JL (2006). B and T lymphocyte attenuator-mediated signal transduction provides a potent inhibitory signal to primary human CD4 T cells that can be initiated by multiple phosphotyrosine motifs. J Immunol.

[48] Chemnitz JM, Parry RV, Nichols KE, June CH, Riley JL (2004). SHP-1 and SHP-2 associate with immunoreceptor tyrosine-based switch motif of programmed death 1 upon primary human T cell stimulation, but only receptor ligation prevents T cell activation. J Immunol.

[49] Yokosuka T, Takamatsu M, Kobayashi-Imanishi W, Hashimoto-Tane A, Azuma M, Saito T (2012). Programmed cell death 1 forms negative costimulatory microclusters that directly inhibit T cell receptor signaling by recruiting phosphatase SHP2. J Exp Med.

[50] Le Mercier I, Lines JL, Noelle RJ (2015). Beyond CTLA-4 and PD-1, the Generation Z of Negative Checkpoint Regulators. Frontiers in immunology.

[51] Pfistershammer K, Klauser C, Pickl WF, Stockl J, Leitner J, Zlabinger G, Majdic O, Steinberger P (2006). No evidence for dualism in function and receptors: PD-L2/B7-DC is an inhibitory regulator of human T cell activation. Eur J Immunol.

[52] Derdak SV, Kueng HJ, Leb VM, Neunkirchner A, Schmetterer KG, Bielek E, Majdic O, Knapp W, Seed B, Pickl WF (2006). Direct stimulation of T lymphocytes by immunosomes: virus-like particles decorated with T cell receptor/CD3 ligands plus costimulatory molecules. Proc Natl Acad Sci U S A.

[53] Ratzinger F, Haslacher H, Poeppl W, Hoermann G, Kovarik JJ, Jutz S, Steinberger P, Burgmann H, Pickl WF, Schmetterer KG (2014). Azithromycin suppresses CD4(+) T-cell activation by direct modulation of mTOR activity. Scientific reports.

[54] Leitner J, Kuschei W, Grabmeier-Pfistershammer K, Woitek R, Kriehuber E, Majdic O, Zlabinger G, Pickl WF, Steinberger P (2010). T cell stimulator cells, an efficient and versatile cellular system to assess the role of costimulatory ligands in the activation of human T cells. J Immunol Methods.

[55] Leitner J, Klauser C, Pickl WF, Stockl J, Majdic O, Bardet AF, Kreil DP, Dong C, Yamazaki T, Zlabinger G, Pfistershammer K, Steinberger P (2009). B7-H3 is a potent inhibitor of human T-cell activation: No evidence for B7-H3 and TREML2 interaction. Eur J Immunol.

